# Altered resting-state networks in adolescent non-suicidal self-injury—a graph theory analysis

**DOI:** 10.1093/scan/nsac007

**Published:** 2022-01-28

**Authors:** Ines Mürner-Lavanchy, Julian Koenig, Corinna Reichl, Romuald Brunner, Michael Kaess

**Affiliations:** University Hospital of Child and Adolescent Psychiatry and Psychotherapy, University of Bern, Bern 60 3000, Switzerland; University Hospital of Child and Adolescent Psychiatry and Psychotherapy, University of Bern, Bern 60 3000, Switzerland; Department of Child and Adolescent Psychiatry, Centre for Psychosocial Medicine, University of Heidelberg, Heidelberg 69115, Germany; Department of Child and Adolescent Psychiatry, Psychosomatics and Psychotherapy, Faculty of Medicine and University Hospital Cologne, University of Cologne, Cologne 50931, Germany; University Hospital of Child and Adolescent Psychiatry and Psychotherapy, University of Bern, Bern 60 3000, Switzerland; Clinic and Policlinic of Child and Adolescent Psychiatry, Psychosomatics and Psychotherapy, University of Regensburg, Regensburg 93053, Germany; University Hospital of Child and Adolescent Psychiatry and Psychotherapy, University of Bern, Bern 60 3000, Switzerland; Department of Child and Adolescent Psychiatry, Centre for Psychosocial Medicine, University of Heidelberg, Heidelberg 69115, Germany

**Keywords:** non-suicidal self-injury, functional connectivity, resting-state fMRI, adolescents, graph theory

## Abstract

Non-suicidal self-injury (NSSI) is a highly prevalent transdiagnostic symptom and risk marker for mental health problems among adolescents. Research on the neurobiological mechanisms underlying NSSI is needed to clarify the neural correlates associated with the behavior. We examined resting-state functional connectivity in *n* = 33 female adolescents aged 12–17 years engaging in NSSI, and in *n* = 29 age-matched healthy controls using graph theory. Mixed linear models were evaluated with the Bayes Factor to determine group differences on global and regional network measures and associations between network measures and clinical characteristics in patients. Adolescents engaging in NSSI demonstrated longer average characteristic path lengths and a smaller number of weighted hubs globally. Regional measures indicated lower efficiency and worse integration in (orbito)frontal regions and higher weighted coreness in the pericalcarine gyrus. In patients, higher orbitofrontal weighted local efficiency was associated with NSSI during the past month while lower pericalcarine nodal efficiency was associated with suicidal thoughts in the past year. Higher right but lower left pericalcarine weighted hubness was associated with more suicide attempts during the past year. Using a graph-based technique to identify functional connectivity networks, this study adds to the growing understanding of the neurobiology of NSSI.

## Introduction

Non-suicidal self-injury (NSSI) is defined as self-injurious behavior in the absence of suicidal intent. In adolescents, the prevalence for single events of NSSI in population-based samples according to a meta-analysis is 17.2% (Swannell *et al.*, 2014). The rising awareness and clinical as well as scientific interest in the phenomenon of NSSI has been reflected by the introduction of the NSSI disorder to the 5th version of the Statistical and Diagnostic Manual of Mental Disorders (DSM-5) under section III, as a research diagnosis requiring further study (American Psychiatric Association, 2013). Criterion A of NSSI disorder is met when a person engages in self-injury without suicidal intent on 5 or more days within the past year. Its prevalence has been found to be 4% in adolescent non-clinical samples (Plener *et al.*, 2016) and around 50% among adolescent in-patient samples (Glenn and Klonsky, 2013; Groschwitz *et al.*, 2015). Previous NSSI history has shown to predict future NSSI and suicide attempts (Asarnow *et al.*, 2011) and increase the risk for suicide (Hawton *et al.*, 2015). NSSI often occurs in the context of psychiatric conditions and is one of the strongest predictors of borderline personality disorder (BPD) development (Ghinea *et al.*, 2019). Importantly, recent research suggests that NSSI is to be seen as a transdiagnostic risk marker or precursor of psychopathology in general (Ghinea *et al.*, 2020).

Up to date, little is known about the neurobiological basis of NSSI. According to the proposed ‘model of distal and proximal trait biology as well as biological states’, the neurobiology of NSSI can be described by ‘distal biological traits’ representing genetic or risk factors predicting the development of NSSI, ‘proximal biological traits’ defined as underlying biological alterations observed in individuals engaging in NSSI and ‘biological states’ immediately causing or precipitated by NSSI, such as reactivity of endocrine and physiological systems (Kaess *et al.*, 2021). In the present study, we aim to contribute to the understanding of proximal traits, namely brain functional alterations in patients with NSSI disorder.

Most studies investigating neurobiological alterations in patients engaging in NSSI used task-based paradigms to examine brain activation: young female adults with NSSI showed higher activation of orbitofrontal brain areas during a gambling task (Vega *et al.*, 2018), decreased prefrontal cortex (PFC) activation during an interference task (Dahlgren *et al.*, 2018) and heightened amygdala responses to negative emotional stimuli (Niedtfeld *et al.*, 2010). Adolescents engaging in NSSI further showed increased activation of the medial PFC and the ventrolateral PFC in a social exclusion paradigm (Groschwitz *et al.*, 2016) and increased amygdala activity in response to negative stimuli (Plener *et al.*, 2012) compared to healthy controls. Studies adopting a network perspective using measures of functional connectivity in adults found diminished connectivity between the left orbitofrontal cortex (OFC) and the right parahippocampal gyrus during a gambling task (Vega *et al.*, 2018) and enhanced amygdala-frontal cortex connectivity following the presentation of negative stimuli (Niedtfeld *et al.*, 2010). In youth with NSSI (*n* = 13), reduced functional connectivity between right OFC and anterior cingulate cortex (ACC) was found during experimental pain administration (Osuch *et al.*, 2014).

More recent studies have begun to elucidate neural circuits implicated in NSSI using resting-state functional magnetic resonance imaging (rsfMRI) to determine resting-state functional connectivity (RSFC). Female adolescents with NSSI demonstrated higher seed-based RSFC between right amygdala and dorsal ACC as well as supplementary motor area (SMA) and lower negative amygdala RSFC with lateral occipital and angular, frontal pole, as well as inferior and middle temporal regions than healthy controls. Further, NSSI had lower positive left amygdala RSFC with dorsal ACC and SMA (Westlund Schreiner *et al.*, 2017). In a similar seed-based approach, adolescents engaging in NSSI showed reduced amygdala RSFC with the ACC, subcallosal cortex, paracingulate gyrus, right planum temporale and right insula as well as between the medial PFC and pre- and postcentral gyri and left insula, compared to healthy controls (Santamarina-Perez *et al.*, 2019).

Disturbed or altered functional brain networks related to psychiatric disorders or symptoms can be described using graph theory. Graph theoretical analysis of RSFC networks has the potential to quantify global and local patterns of brain connectivity, obtained through rsfMRI. These graph-based analyses yield useful network metrics including network efficiency and organization and illustrate key regions within the network (Bullmore and Sporns, 2009). Complex brain networks are abstractly described as graphs that are composed of nodes, which represent brain regions, linked together by edges that denote connections (Sporns, 2014). When applied to rsfMRI, the nodes identified by graph theory represent brain regions, while the edges represent functional connections corresponding to magnitudes of temporal correlations in blood-oxygen-level-dependent activity between pairs of brain regions (Rubinov and Sporns, 2010). Compared to other methods, graph-based network analysis has the potential to reveal meaningful information about the topological architecture of human brain networks, such as small-worldness, modular organization, and highly connected or centralized hubs. To the best of our knowledge, this approach has not yet been applied to RSFC networks in NSSI.

In the present study, we aimed to investigate brain networks of RSFC in adolescents engaging in NSSI and healthy controls using graph-based analyses. The first aim of the present study was to investigate differences in RSFC between adolescents engaging in NSSI and healthy controls using graph theory on a global brain level. The second aim was to examine group differences on a regional brain level across 76 regions of interest (ROIs) spanning the whole cortex. The third aim of the study was to explore dimensional associations between graph-based RSFC measures and clinical characteristics of NSSI severity, suicidality, depressive symptoms and BPD criteria in adolescent patients with NSSI.

## Methods and materials

### Participants

The study procedure has been described in detail elsewhere (Reichl *et al.*, 2016, 2019; Ando *et al.*, 2018). Adolescents between 12 and 17 years with NSSI disorder according to DSM-5 were recruited from the specialized outpatient clinic for adolescent risk-taking and self-harm behavior (AtR!Sk; ‘Ambulanz für Risikoverhalten & Selbstschädigung’ (Kaess *et al.*, 2017)) and the inpatient units at the Clinic of Child and Adolescent Psychiatry, University Hospital Heidelberg, Germany. Healthy participants who were age- and sex-matched who had neither received lifetime psychiatric diagnosis nor undergone psychiatric treatment or engaged in NSSI were recruited via public advertisement. Adolescents with acute psychotic symptoms, acute suicidality requiring immediate intervention and poor knowledge of the German language were not included. Informed and written consent was obtained from participants and their parents/caregivers. The study was approved by the institutional ethics committee of the University of Heidelberg (S-449/2013) and was performed in accordance with the Declaration of Helsinki.

### Procedure

Included participants underwent a clinical appointment and an MRI exam at the University Hospital Heidelberg. Participants who were pregnant, claustrophobic, had metal implants or a history of brain injury were not included in the MRI part of the study.

### Clinical assessment

The sociodemographic information obtained during the clinical assessment included sex, date of birth, medication and drug use. Handedness was assessed using the Edinburgh Handedness Inventory (Oldfield, 1971). All clinical interviews were performed by clinicians trained in the field of child and adolescent psychiatry.

#### NSSI and suicidal thoughts, plans and attempts

NSSI was assessed using the German version (Fischer *et al.*, 2014) of the Self-Injurious Thoughts and Behavior Interview (SITBI) (Nock *et al.*, 2007). The semi-structured interview assesses the presence, frequency and characteristics of a variety of thoughts and behaviors of NSSI as well as suicidal thoughts, gestures, plans and attempts. Previous research reported good reliability estimates for the German version of the SITBI and good convergent validity in relation to an established questionnaire of self-harming behavior (Fischer *et al.*, 2014).

#### Psychiatric diagnoses

The Mini-International Neuropsychiatric Interview for Children and Adolescents (M.I.N.I.-KID 6.0), (Sheehan *et al.*, 2010), a semi-structured interview for the assessment of axis I psychiatric disorders according to DSM-IV and ICD-10, was used to assess psychiatric diagnoses of the NSSI group. Additionally, BPD criteria were assessed with the respective module of the German version of the Structured Clinical Interview for DSM-IV Personality Disorders (SKID II) (Fydrich *et al.*, 1997). Depressive symptoms were assessed with the German version of the Beck Depression Inventory II (Hautzinger *et al.*, 1995).

### Neuroimaging

The mean number of days between the clinical assessment session and the imaging session was 26.8 days (s.d. = 28.2) for adolescents engaging in NSSI and 28.8 days (s.d. = 32.2) for healthy controls. Participants were scanned using a Magnetom TRIO (Siemens, Erlangen, Germany) 3 Tesla scanner with a 32-channel-head coil. Anatomical T1-weighted images were acquired in the sagittal plane (192 slices, 1 mm slice thickness, 1 × 1 mm^2^ in-plane resolution, echo time = 2.52 ms, repetition time = 1900 ms, flip angle = 9°). T2*-weighted echo planar images were collected during a resting state (echo time (TE) = 27 ms, repetition time (TR) = 2650 ms, field of view (FoV) = 220 mm, flip angle = 90°, slices = 45), during which participants were asked to keep their eyes open.

### Image processing

Standard preprocessing of rsfMRI data was carried out using FMRIB’s Software Library (FSL 5.0, Oxford, 2012) and included motion correction with MCFLIRT, brain extraction using BET, slice time correction, spatial smoothing with a Gaussian kernel of full-width-half-maximum 6 mm and high-pass temporal filtering. Functional images were registered linearly with the processed brain extracted images using the FMRIB linear image registration tool and then co-registered non-linearly with MNI standard space using FNIRT. Automated structural segmentation of the T1-weighted images was carried out using FreeSurfer version 6.0. (Reuter *et al.*, 2010) with the Desikan–Killiany–Tourville atlas (Klein and Tourville, 2012). FreeSurfer performs volume-based segmentations where labeling of brain structures is performed by registration of T1-weighted images to MNI305 space, followed by segmentation based on both a subject-independent probabilistic atlas and subject-specific measured values, and then classification of each voxel to a label. Details of FreeSurfer segmentation have been described previously (Fischl *et al.*, 2002, 2004). After segmentation, all images used in the analyses were visually checked for quality by trained researchers.

### Connectivity network

Network construction was performed in R version 3.6.3 (https://www.r-project.org/), using the packages igraph v1.2.1 and brainGraph v2.4.0 (Csardi and Nepusz, 2006; Watson *et al.*, 2018). A total of 76 subject-specific ROIs derived from FreeSurfer were used as nodes. Given the sparse existing literature on RSFC in NSSI, we included ROIs spanning the whole cortex in order to provide the full picture of potential group differences in RSFC. A connectivity matrix consisting of all nodes was created, where the connection of two nodes is represented by the squared partial correlation of the time series with the time series of all other nodes (to control for indirect connections between the connections of interest) and cerebrospinal fluid and white matter partialed out. Graph metric data were extracted and saved for further statistical analyses.

### Graph theory metrics

A range of metrics have been developed that are designed to capture different organizational properties of a network (Rubinov and Sporns, 2010). In this study, we explored ‘global’, i.e. whole brain as well as ‘regional’ (nodal) connectivity using standard graph theory metrics as implemented in the brainGraph package (Csardi and Nepusz, 2006; Watson *et al.*, 2018). Measures of unweighted (binary, value of 0 = absence or 1= connection) and weighted graphs (associated with a real number, which indicates strength of the connection) were included. Measures are listed in detail in the Supplementary Material.

### Statistical analyses

All network measures and clinical variables were *z*-transformed to enable comparison of regression coefficients. For the first aim, regression models including global network measures as a dependent variable and group (NSSI *vs* healthy control) as an independent variable (M1) were compared to null models (M0) containing only the dependent variable as a regressor. For aim two, three mixed linear regression models were compared: M0 included the regional network measure and the factor of hemisphere (left *vs* right) as a covariate; model M1 additionally included the factor group as an independent variable; model M2 further included the interaction of hemisphere and group to investigate potential effects of laterality. For the third aim, those measures/ROIs showing ‘strong evidence’ for group differences were subjected to further analysis, in patients only. Again, three mixed linear models were compared, where M0 was compared to M1 containing the clinical variable of interest (with hemisphere as a covariate in M0 and M1) and M2 additionally containing the interaction between the clinical variable and hemisphere. Clinical variables included in these analyses were as follows: suicidal thoughts (frequency in days, lifetime, last year and last month), suicidal plans (frequency in days, lifetime and last year), suicide attempts (frequency in attempts, lifetime and last year), acts of NSSI (frequency in days, lifetime, last year, last month and last week), number of BPD criteria and depression severity. A number of variables were not assessed further due to minimal variance (suicidal thoughts last week; suicidal plan last month, last week; suicide attempts last month and last week). Outliers at more than 3 s.d. above the group mean were excluded from these analyses. As most outliers were clinically plausible, additional analyses with less stringent outlier removal are presented alongside the main results (only one extreme data point for NSSI during the past month was removed, while all other data points were retained in the analyses). Instead of using *P*-value statistics, requiring further adjustment for multiple comparisons, models were compared using the Bayes Factor (BF), where a BF between 3 and 20 can be seen as ‘positive evidence’ and a BF larger than 20 is considered ‘strong evidence’ (Raftery, 1995). Model selection based on Bayesian parameter estimation has several advantages over model selection based on *P*-values (Freedman, 1983; Freedman *et al.*, 1988; Miller, 1990). While Bayesian model selection does not require *P*-values for statistical comparison, corresponding *P*-values have been included in the ‘Results’ section for readers more familiar to the frequentist statistical approach. Statistical analyses were performed using Stata (Version 16; StataCorp LP, College Station, TX, USA). Among the wealth of network measures, it has not been established yet, which measures are most informative for the analysis of (functional) brain networks (Liu *et al.*, 2017). Therefore, we have chosen an explorative approach including all available network measures from the software used. Results with strong evidence (BF > 20) will be emphasized in the main manuscript, while results with ‘positive evidence’ (BF 3–20) will be reported in summary to provide the full picture of results, and details are given in the Supplementary Material.

## Results

### Demographic and clinical characteristics of participants

Out of *n* = 67 adolescents engaging in NSSI and *n* = 47 healthy controls, *n* = 36 youth with NSSI and *n* = 31 controls underwent MRI. Of these, *n* = 1 patient had a space-consuming lesion, which led to unusable T1 images, and *n* = 2 participants had rsfMRI data that were not usable. For the purpose of the present study, another *n* = 2 male patients with NSSI were excluded from further analyses due to the known effects of sex on brain development, resulting in a study sample of *n* = 33 adolescents with NSSI and *n* = 29 healthy controls.

Demographic and clinical characteristics for both groups are detailed in Table 1. Adolescents were comparable in age, handedness and body mass index between groups. Regarding education, *n* = 18 adolescents engaging in NSSI and *n* = 21 controls attended the ‘Gymnasium’ (graduation qualifies for university entrance); *n* = 4 adolescents from the NSSI group and *n* = 7 controls attended or completed the ‘Realschule’ (secondary school level certificate) and *n* = 7 from the NSSI group, and *n* = 1 controls attended or completed the ‘Hauptschule’ (9 years of elementary school). Within the NSSI group, 12% reported regular use of medication and 30% reported substance use, whereas this was the case in 3.5% (*n* = 1) of the control group.

**Table 1.** Participant characteristics

	NSSI *n* (%)/mean (s.d.)	Control *n* (%)/mean (s.d.)
*N*	33	29
Age (years)	15.84 (1.33)	16.02 (1.12)
Right handedness	30 (91)	27 (93)
Body mass index	21.89 (3.48)	20.73 (2.46)
Regular use of medication	4 (12)	1 (4)
Substance use^a^	5 (15)	1 (4)
Acts of NSSI last year	66.39 (76.80)	0
Acts of NSSI last month	3.39 (5.45)	0
Suicidal thoughts last year	5.50 (6.46)	0
Suicidal thoughts last month	1.13 (0.99)	0
Suicide attempts lifetime	1.83 (1.38)	0
Suicide attempts last year	1.00 (0.77)	0
Number of BPD diagnostic criteria	4.24 (2.33)	0.07 (0.26)
BPD diagnosis	14 (42)	0 (0)
Depressive symptoms	29.67 (15.15)	4.07 (3.59)
Depression diagnosis	25 (76)	0 (0)

*Notes*: NSSI = non-suicidal self-injury; BPD = borderline personality disorder.

a On 3 or more days in the last 3 months.

In the NSSI group, the mean frequency of NSSI was 66.39 (s.d. = 76.80) during the last year and 3.39 (s.d. = 5.45) during the last month. The mean reported number of suicide attempts was 1.83 (s.d. = 1.38) across the lifetime and 1.00 (s.d. = 0.77) during the last year.

### Group differences in functional connectivity

#### Global network level

On the global network level, there was no strong evidence that groups differed for any of the network measures. However, there was positive evidence that ‘weighted characteristic path length’ was longer in the NSSI group (M_NSSI_ = 34.9, s.d. = 6.7; M_control_ = 30.8, s.d. = 4.9; BF = 4.5) and that the ‘weighted number of hubs’ (vertices with hubness score ≥2) was smaller in the NSSI group compared to healthy controls (M_NSSI_ = 13.1, s.d. = 2.4; M_control_ = 14.5, s.d. = 1.7; BF = 3.1) (see Supplementary Table S1).

#### Regional network (vertex) level

**Fig. 1.**
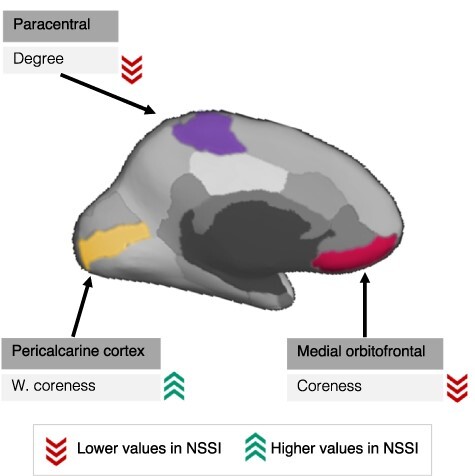
Regional group differences in network measures between adolescents engaging in NSSI *vs* healthy controls.

*Notes*: ‘coreness’ = referring to the k-core, which is the largest subnetwork comprising nodes of degree at least *k*. It reflects membership in sub-networks of multiple highly interconnected nodes. ‘W. (= weighted) coreness’* *= refers to the s-core, the largest subnetwork comprising nodes of strength at least *s*. ‘Degree = number of connections of a node’. Regions of interest are illustrated on the left hemisphere, but models were calculated for left and right hemispheres (with hemisphere as a covariate).

For the regional network measures, there was strong evidence (BF > 20) of group differences between adolescents engaging in NSSI and controls in three medial frontal and occipital ROIs (Table 2, Figure 1). Medial OFC ‘coreness’, which represents membership in sub-networks of multiple highly interconnected nodes (Hagmann *et al.*, 2008), was lower in the NSSI than in the control group. In contrast, pericalcarine ‘weighted coreness’ was higher in the NSSI group than in the control group. Finally, paracentral ‘degree’, denoting the number of edges, or connections of a vertex, was lower in the NSSI group.

**Table 2.** Regression coefficients of models showing strong evidence of group differences in regional functional connectivity (BF > 20)

ROI	Network measure	NSSI mean (s.d.)	Controls mean (s.d.)	Coefficient (95 % CI)	*P*	BF
Medial orbitofrontal	Coreness	1.55 (0.56)	2.16 (0.92)	−0.76 (−1.18, −0.34)	<0.001	25.4
Paracentral	Degree	2.23 (1.20)	3.17 (1.65)	−0.51 (−0.80, −0.22)	0.001	21.0
Pericalcarine cortex	W. coreness	51.05 (12.83)	38.0 (16.3)	0.65 (0.30, 1.01)	<0.001	36.0

*Notes*: Results from models with BF (= Bayes Factor) >20 are shown. NSSI = non-suicidal self-injury, CI = confidence interval. Coefficients and 95% CI refer to *z*-standardized variables. W. = Weighted measure includes information about connection strength (as opposed to unweighted measures, which refer to binary graph (connection absent *vs* present)).

Further, there was positive evidence (BF 3–20) of group differences in a number of regional network measures mainly in frontal ROIs (paracentral, precentral, caudal middle frontal and pars opercularis, known as SMA), where network measures indicated longer path lengths, lower ‘degrees’ (number) and ‘strengths’ of connections, lower ‘local efficiency’ and lower ‘coreness’ (Supplementary Table S2).

### Associations between functional connectivity and clinical characteristics in patients

**Fig. 2.**
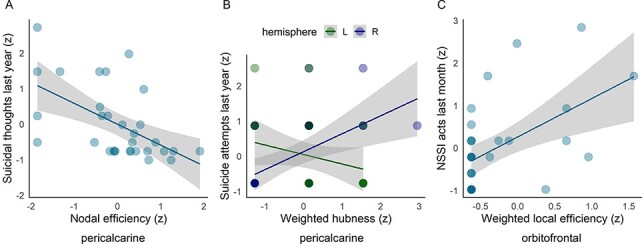
Associations between graph-based measures of regional functional connectivity and clinical characteristics in patients engaging in NSSI.

*Notes*: Associations of graph-based measures with (A) suicidal thoughts, (B) suicide attempts and (C) acts of NSSI. Results from models with BF (= Bayes Factor) >20 are shown. Each individual measurement is represented by a circle, lines represent linear regression coefficient and grey shaded area represents the standard error. Circles appear darker when multiple measurements are overlaid.

Associations between regional network measures and clinical characteristics for those ROIs which showed strong evidence of group differences between NSSI and controls (BF > 20), namely medial OFC, paracentral and pericalcarine gyrus, are reported in Table 3 and Figure 2. Associations with positive evidence (BF 3–20) in these ROIs are reported in the Supplementary Material (Supplementary Table S3).

**Table 3.** Regression coefficients of models showing strong evidence of associations between regional functional connectivity and clinical variables (BF > 20)

Analyses with conservative outlier management						
Clinical characteristic		ROI	Network measure	Coefficient (95 % CI)	*P*	BF
Suicidal thoughts	Past year	Pericalcarine	Nodal efficiency	−0.52 (−0.80, −0.25)	<0.001	37.2
Suicide attempts	Past year	Pericalcarine	W. hubness^a^	0.77 (0.43, 1.11)	<0.001	59.5
NSSI acts	Past month	Medial orbitofrontal	W. local efficiency	0.31 (0.14, 0.49)	<0.001	25.2
**Analyses with less conservative outlier management**						
Suicidal thoughts	Lifetime	Medial orbitofrontal	W. transitivity	0.30 (0.19, 0.41)	<0.001	1136.2
Suicidal thoughts	Lifetime	Medial orbitofrontal	Transitivity	0.32 (0.21, 0.42)	<0.001	16 256.9
Suicidal thoughts	Lifetime	Medial orbitofrontal	W. local efficiency	0.30 (0.13, 0.47)	<0.001	22.6
Suicidal thoughts	Lifetime	Medial orbitofrontal	Local efficiency	0.33 (0.22, 0.45)	<0.001	10 275.4
NSSI acts	Past month	Medial orbitofrontal	W. local efficiency	0.31 (0.14, 0.49)	<0.001	25.2
NSSI acts	Past week	Medial orbitofrontal	W. transitivity^a^	0.42 (0.28, 0.57)	<0.001	5422.9

*Notes*: Associations between network measures in ROIs where there was strong evidence of group differences in primary analyses. Results from models with BF (= Bayes Factor) > 20 are shown. CI = confidence interval. W. = Weighted measure: includes information about connection strength (as opposed to unweighted measures, which refer to binary graph (connection absent *vs* present)).

a Interaction effect of hemisphere by suicide attempts. Conservative outlier management: outliers ±3 s.d. removed. Less conservative outlier management: clinically plausible but high values included, except for one implausible value in NSSI acts past month.

Strong associations between regional network measures and clinical characteristics were only found in OFC and pericalcarine cortex. The more days with suicidal thoughts patients reported during the last year, the lower was ‘nodal efficiency’ in the pericalcarine gyrus. Further, a higher number of reported suicide attempts last year was associated with higher ‘weighted hubness’ in the right pericalcarine gyrus, but with lower ‘weighted hubness’ in the left pericalcarine gyrus. Finally, the higher the number of NSSI acts in the last month, the higher the ‘weighted local efficiency’ in the medial OFC. Additional analyses with less conservative outlier management further showed evidence for strong associations between lifetime suicidal thoughts and medial OFC ‘transitivity’ and ‘local efficiency’ as well as NSSI acts within the past week and medial OFC ‘weighted transitivity’. For these measures, higher severity in clinical characteristics was related to higher ‘transitivity’ and ‘local efficiency’ (Table 3 and Supplementary Figure S1).

Positive evidence of associations between medial OFC measures of higher ‘local efficiency’ and ‘clustering’ but worse integration (i.e. longer ‘characteristic path lengths’) was found in relation to a higher number of suicidal thoughts, plans, suicide attempts and acts of NSSI (Supplementary Table S3). Moreover, results indicated some evidence for higher paracentral ‘participation coefficients’ (indicating that this region has most connections not within the community but with different communities) and lower ‘degree’ (number of links) associated with more suicidal thoughts, plans and acts of NSSI. Finally, lower pericalcarine ‘nodal efficiency’ and ‘average nearest neighbor degree’, which quantifies the average degree of connections of a node, and lower ‘weighted hubness’ were associated with more suicidal thoughts and attempts.

## Discussion

This study adds to the limited understanding of brain functional connectivity associated with adolescent NSSI by applying a graph-based network approach. To our knowledge, this is the first study to apply graph theory to investigate the neurobiological underpinnings of NSSI. On a global brain level, adolescents with NSSI exhibited longer ‘shortest characteristic path lengths’ indicating less efficient information transfer. A smaller ‘number of hubs’ further reflects that there are fewer areas of highly interconnected nodes, which are central for information integration, in adolescents with NSSI than in controls. Of note, none of the global group differences described above showed strong evidence (all BF > 3 but < 20). On a regional level, youth with NSSI demonstrated lower ‘coreness’ in the OFC in comparison with healthy controls. ‘Coreness’ is a measure denoting the largest subnetwork of a graph with the highest number of connections in comparison to the surrounding nodes. A lower ‘coreness’ might therefore indicate a less prominent role of the OFC and lower orbitofrontal RSFC in NSSI youths than in controls. This is in line with findings from task-based fMRI, where blunted reward anticipation in the OFC was demonstrated in self-injuring adolescents (Sauder *et al.*, 2016), which has consistently been shown in disorders related with low impulse control and anhedonia or depression. Reduced connectivity between the left OFC and the right parahippocampal gyrus was found in adults with BPD during a gambling task (Vega *et al.*, 2018), and reduced connectivity between right OFC and ACC was found during experimental pain administration in adolescents with NSSI (Osuch *et al.*, 2014). In line with the alterations shown in task-based fMRI studies, our finding of lower ‘coreness’ in youth with NSSI might suggest a deficit of the orbitofrontal regulation of emotional responses and impulsivity normally executed by the OFC on the amygdala. Further, with the OFC involved in evaluating reward values and outcomes (Stalnaker *et al.*, 2015), our findings add evidence of the importance of the brain’s reward circuitry in the context of NSSI (Brunner *et al.*, 2010; Sauder *et al.*, 2016; Poon *et al.*, 2019).

We also found a lower ‘degree’ in the paracentral gyrus, which indicates fewer connections in this region in adolescents engaging in NSSI compared to healthy controls. The paracentral lobule is known for its sensory and motor control of the lower extremities. More recently, it has been implicated in emotion processing in depressive patients with histories of stressful life events (Li *et al.*, 2016). A structural MRI study found that greater impulsivity in depressed adolescents with a history of suicide attempts was related to cortical thickness of the paracentral lobule (Fradkin *et al.*, 2017). To the best of our knowledge, however, the role of the paracentral lobule as a possible correlate of impulsivity or action planning has not previously been discussed in the context of NSSI.

Weaker group differences in several frontal ROIs, namely precentral, caudal middle frontal and pars opercularis, known as SMA, were found, with longer ‘path lengths’, lower ‘degrees (number)’ and ‘strengths’ of connections, lower ‘local efficiency’ and lower ‘coreness’ in patients with NSSI than in controls. These differences seem again to fit the idea of an impaired frontal top-down control that is suggested to play an important role in both emotional dysregulation and impulsive behavior (Etkin *et al.*, 2015). However, given that evidence was positive but not strong, results may need replication in larger samples.

Finally, adolescents with NSSI demonstrated higher ‘weighted coreness’ in the pericalcarine cortex, which suggests a subnetwork of strong connections in this region compared to the healthy control group. As part of the secondary visual cortex, this region is generally implicated in visual information processing. Interestingly, it has been related to the processing of self-related information during social interaction in a group of NSSI and healthy adolescents; however, this effect was not specific to the NSSI group (Perini *et al.*, 2019). While the role of visual processing has been demonstrated in NSSI in the context of face recognition (Seymour *et al.*, 2016), the potential importance of the pericalcarine cortex for NSSI needs to be clarified by further studies. Overall, our results suggesting worse information transfer in youth engaging in NSSI are in line with findings from structural MRI, which indicate widespread white matter microstructural deficits in adolescents with NSSI (Westlund Schreiner *et al.*, 2019a).

A further aim of this study was to determine whether RSFC was related to specific clinical characteristics in the NSSI group. Interestingly, in our study, more acts of NSSI in the last month were related to a higher orbitofrontal ‘weighted local efficiency’. In the context of generally lower OFC efficiency in youth with NSSI (as discussed above), this suggests that the act of NSSI might serve as a compensatory mechanism for emotional dysregulation potentially due to limbic hyperarousal (Plener *et al.*, 2012). This finding is further accompanied by the weaker findings of higher OFC clustering, but worse integration (longer path lengths) associated with more suicidal thoughts, plans, attempts and acts of NSSI and might indicate ‘overactivity’ within the OFC but simultaneously worse information transfer with other regions, such as the amygdala. Similarly, despite higher pericalcarine ‘weighted coreness’ in the NSSI group, those youths who reported more suicidal thoughts in the previous year demonstrated lower ‘nodal efficiency’ in this region. Moreover, fewer connections (‘degree’) and hubs were associated with more suicidal thoughts and attempts in this region. Given that a minority of the studies have demonstrated associations with clinical outcomes in addition to group differences, our findings are difficult to integrate in the current literature. Additionally, our results show that the association between network measures and clinical outcomes may in some brain regions depend on the hemisphere. Our findings therefore reflect that the associations between network measures and clinical characteristics are not straightforward and need to be replicated in larger samples.

A few limitations merit comment. First, the neurobiological and neurophysiological processes underlying graph metrics are still unknown, and these metrics may vary depending on methodological decisions such as the parcellation scheme (Farahani *et al.*, 2019). We have limited our analyses addressing clinical correlates to regions that showed differences between groups. However, RSFC in other brain regions not further examined in these analyses might show meaningful associations with clinical symptoms. For example, the salience network, the default mode network and the central executive network have been involved in the acute stress response, which might contribute to psychopathology when it is inadequate, excessive or prolonged (van Oort *et al.*, 2017). Second, the clinical variables were assessed based on retrospective interviews. More ecologically valid approaches such as ecological momentary assessment with repeated sampling of patients’ experiences or mood have the potential to reduce retrospective bias (Solhan *et al.*, 2009). Third, RSFC changes with continuing maturation of the functional and structural networks in childhood and well into adolescence (Fair *et al.*, 2009; Huang *et al.*, 2013). Therefore, longitudinal studies investigating RSFC networks are needed to elucidate whether processes of neuromaturation play an important role in the pathways to risk behaviors or might reveal neural circuits potentiating the risk for self-injurious behaviors. In particular, the changes related to emotion processing and executive functions taking place during adolescence and puberty (Blakemore, 2008) are likely to be intertwined with the emergence of NSSI. Finally, we acknowledge that first and foremost, the results from this study add to the basic understanding of the neurobiology of NSSI and have limited clinical implications. Nevertheless, critical alterations in functional and structural brain characteristics have previously been associated with pharmacological and psychotherapeutic interventions (Quidé *et al.*, 2012; Cullen *et al.*, 2016). A first study in adolescents with NSSI found stronger negative amygdala-prefrontal connectivity associated with greater post-treatment improvement in NSSI after Dialectical Behavior Therapy for Adolescents and treatment as usual (both treatment arms had to be combined to increase power to investigate RSFC connectivity correlates of psychological treatment) (Santamarina-Perez *et al.*, 2019). Potentially, a better understanding of the neurobiological underpinnings of NSSI may in the future help to identify neurobiological treatment targets and thereby advance treatment development (Westlund Schreiner *et al.*, 2019b).

## Conclusion

The current study sheds new light on RSFC associated with NSSI. Using graph theory, we have demonstrated that adolescents engaging in NSSI show altered topological organization of functional networks during rest. The pattern of results was less clear for the clinical associations in patients, with higher and lower network efficiency relating to worse clinical outcomes depending on the brain region and hemisphere. By means of cutting-edge network science and a relatively large sample size, our study adds to elucidating the neurobiological concomitants of adolescent NSSI.

## Supplementary Material

nsac007_Supp
