# Supplementary material for: Altered resting-state networks in adolescent non-suicidal self-injury—a graph theory analysis
Source: Soc Cogn Affect Neurosci. 2022 Jan 28;17(9):819–27. doi: 10.1093/scan/nsac007 (PMC9433841; doi:10.1093/scan/nsac007)
Supplement: nsac007_Supp [file nsac007_supp.zip › scan-21-076-File007.docx]

# **Supplementary material**

**List of global and regional RSFC network measures**

*Global* measures: *global efficiency, weighted global efficiency, mean local efficiency, weighted mean local efficiency, characteristic path length, weighted characteristic path length, diameter, weighted diameter, modularity (Louvain), weighted modularity, number of hubs, weighted number of hubs, clustering coefficient, density, site of the largest connected component, number of triangles in the graph, transitivity, degree assortativity, average vertex strength*, and *maximal vulnerability across vertices*.

*Regional* measures: *local efficiency, weighted local efficiency, nodal efficiency, weighted nodal efficiency, average shortest path lengths, weighted average shortest path lengths, degree, strength* (the weighted counterpart of *degree*), *average nearest neighbor degree, average nearest neighbor strength, eccentricity, hubness, weighted hubness, coreness, weighted coreness, gateway coefficient, weighted gateway coefficient, participation coefficient, weighted participation coefficient, transitivity, weighted transitivity, vulnerability, within-module degree z-score, weighted within-module degree z-score, betweenness centrality, eigenvector centrality,* and *leverage centrality*.

**Supplementary Table 1.** Group differences of global network measures.

| Network measure | NSSI  mean (SD) | Controls  mean (SD) | Coefficient (95 % CI) | p | BF |
| --- | --- | --- | --- | --- | --- |
| clustering coefficient | 0.19 (0.05) | 0.20 (0.61) | -2.97e^-16^ (-0.25, 0.25) | 1 | 0.2 |
| global efficiency | 0.21 (0.06) | 0.24 (0.07) | 1.54e^-16^ (-0.25, 0.25) | 1 | 0.5 |
| weighted global efficiency | 0.03 (0.01) | 0.04 (0.01) | -9.47e^-16^ (-0.25, 0.25) | 1 | 0.6 |
| mean local efficiency | 0.16 (0.06) | 0.19 (0.08) | -6.84e^-16^ (-0.25, 0.25) | 1 | 0.3 |
| weighted mean local efficiency | 0.03 (0.01) | 0.04 (0.02) | -1.25e^-16^ (-0.25, 0.25) | 1 | 0.4 |
| characteristic path length | 4.88 (0.98) | 4.34 (0.68) | 0.61 (0.12, 1.10) | 0.015 | 2.8 |
| weighted characteristic path length | 34.85 (6.66) | 30.78 (4.94) | 0.65 (0.17, 1.13) | 0.009 | 4.5 |
| diameter | 12.12 (3.14) | 10.44 (2.13) | 0.59 (0.10, 1.1) | 0.019 | 2.3 |
| weighted diameter | 86.11 (19.64) | 75.00 (15.43) | 0.60 (0.11, 1.10) | 0.017 | 2.4 |
| modularity (Louvain) | 0.66 (0.06) | 0.63 (0.08) | 7.65e^-16^ (-0.25, 0.25) | 1 | 1.0 |
| weighted modularity | 0.77 (0.05) | 0.74 (0.06) | 0.53 (0.03, 1.02) | 0.037 | 1.2 |
| number of hubs | 14.94 (1.71) | 14.86 (1.53) | 5.12e^-16^ (-0.25, 0.25) | 1 | 0.1 |
| weighted number of hubs | 13.12 (2.37) | 14.48 (1.74) | -0.62 (-1.11, -1.13) | 0.013 | 3.1 |
| density | 0.04 (0.01) | 0.04 (0.01) | -2.3e^-16^ (-0.25, 0.25) | 1 | 0.6 |
| number of triangles in the graph | 20.3 (11.94) | 30.69 (33.94) | 2.62e^-16^ (-0.25, 0.25) | 1 | 0.5 |
| transitivity | 0.19 (0.05) | 0.20 (0.06) | 1.76e^-16^ (-0.25, 0.25) | 1 | 0.2 |
| degree assortativity | 0.26 (0.10) | 0.24 (0.12) | 1.59e^-16^ (-0.25, 0.25) | 1 | 0.2 |
| average vertex strength | 0.58 (0.09) | 0.63 (0.13) | 4.43e^-16^ (-0.25, 0.25) | 1 | 0.5 |
| Vulnerability across vertices | 0.14 (0.08) | 0.11 (0.04) | 3.61e^-16^ (-0.25, 0.25) | 1 | 0.8 |

Note. SD = standard deviation, BIC = Bayesian Information Criterion, M0 = Null model, M1 = model including group as factor, BF = Bayes Factor.

**Supplementary Table 2.** Regression coefficients of models showing positive evidence of group differences in regional functional connectivity (BF 3 - 20)

| ROI | Network measure | NSSI mean (SD) | Controls mean (SD) | Coefficient (95 % CI) | p | BF |
| --- | --- | --- | --- | --- | --- | --- |
| Paracentral | W. average nearest neighbor strength | 2.34 (1.23) | 3.22 (1.54) | -0.48 (-0.83, -0.14) | 0.006 | 3.1 |
|  | Betweenness centrality | 44.15 (65.92) | 87.59 (110.91) | -0.31 (-0.54, -0.09) | 0.007 | 3.2 |
|  | Gateway coefficient | 0.10 (0.17) | 0.23 (0.25) | -0.53 (-0.85, -0.20) | 0.001 | 10.0 |
|  | Participation coefficient | 0.07 (0.17) | 0.19 (0.24) | -0.48 (-0.79, -0.17) | 0.002 | 7.0 |
| Precentral | W. average shortest path lengths | 31.09 (8.73) | 25.63 (6.67) | 0.42 (0.15, 0.69) | 0.002 | 6.6 |
|  | Average shortest path lengths | 4.49 (1.24) | 3.79 (0.94) | 0.40 (0.11, 0.68) | 0.006 | 3.2 |
|  | eccentricity | 9.02 (2.70) | 7.28 (2.00) | 0.52 (0.19, 0.86) | 0.002 | 7.3 |
| Postcentral | W. average shortest path lengths | 31.24 (8.78) | 25.91 (6.86) | 0.41 (0.14, 0.69) | 0.003 | 5.2 |
|  | eccentricity | 9.06 (2.62) | 7.47 (2.08) | 0.48 (0.15, 0.81) | 0.004 | 4.3 |
| Pars opercularis | Degree | 3.33 (1.69) | 4.28 (2.13) | -0.51 (-0.87, -0.15) | 0.006 | 3.3 |
|  | Strength | 0.51 (0.23) | 0.67 (0.35) | -0.47 (-0.80, -0.15) | 0.004 | 4.6 |
|  | W. coreness | 26.36 (12.75) | 34.28 (15.49) | -0.39 (-0.67, -0.12) | 0.005 | 3.7 |
|  | W. gateway coefficient | 0.34 (0.28) | 0.50 (0.21) | -0.60 (-0.95, -0.25) | 0.001 | 17.3 |
|  | W. participation coefficient | 0.30 (0.28) | 0.45 (0.21) | -0.60 (-0.96, -0.25) | 0.001 | 16.6 |
|  | W. nodal efficiency | 0.04 (0.01) | 0.04 (0.01) | -0.57 (-.91, -.22) | 0.001 | 10.2 |
|  | Gateway coefficient | 0.29 (0.27) | 0.43 (0.23) | -0.54 (-0.89, -0.19) | 0.003 | 7.1 |
|  | Participation coefficient | 0.25 (0.27) | 0.38 (0.24) | -0.52 (-0.88, -0.15) | 0.005 | 3.9 |
| Caudal middle frontal | Strength | 0.64 (0.32) | 0.86 (0.38) | -0.68, -1.17, -0.20) | 0.005 | 3.4 |
|  | W. coreness | 35.52 (17.47) | 46.72 (14.07) | -0.56 (-0.93, -0.18) | 0.004 | 4.8 |
|  | W. local efficiency | 0.04 (0.06) | 0.08 (0.08) | -0.65, -1.10, -0.20) | 0.004 | 4.1 |
|  | Coreness | 2.17 (0.48) | 2.72 (0.87) | -0.69 (-1.09, -0.29) | 0.001 | 15.3 |
|  | Local efficiency | 0.18 (0.27) | 0.36 (0.30) | -0.62 (-1.03, -0.20) | 0.004 | 4.6 |
| Inferior temporal | W. average nearest neighbor strength | 3.67 (1.40) | 4.65 (1.86) | -0.54 (-0.92, -0.16) | 0.005 | 3.5 |
| Superior temporal | W. average nearest neighbor strength | 3.32 (1.12) | 4.12 (1.55) | -0.46 (-0.77, -0.15) | 0.004 | 4.5 |
| Transverse temporal | W. within-module degree z-score | -0.19 (0.85) | -0.46 (0.70) | 0.54 (0.20, 0.88) | 0.002 | 9.6 |
| Supramarginal | W. average shortest path lengths | 32.77 (7.08) | 27.40 (6.82) | 0.41 (0.16, 0.67) | 0.002 | 8.8 |
|  | Average shortest path lengths | 4.64 (1.07) | 3.94 (0.97) | 0.39 (0.12, 0.67) | 0.005 | 3.9 |
| Pericalcarine | Strength | 0.74 (0.23) | 0.59 (0.22) | 0.45 (0.14, 0.75) | 0.004 | 4.6 |
| Cuneus | Eccentricity | 9.17 (3.25) | 6.93 (2.67) | 0.67 (0.23-1.12) | 0.003 | 5.7 |
| Thalamus | Within-module degree z-score | -0.12 (0.57) | -0.46 (0.71) | 0.39 (0.14, 0.63) | 0.002 | 9.1 |

Note. NSSI = non-suicidal self-injury, SD = standard deviation, CI = confidence interval. Coefficients and 95% CI refer to z-standardized variables. W. = Weighted measures: include information about connection strength; unweighted measures refer to binary graph (connection absent vs. present).

**Supplementary Table 3.** Regression coefficients of models showing positive evidence of associations between regional functional connectivity and clinical variables (BF 3 - 20) in orbitofrontal, paracentral and pericalcarine gyrus.

|  |  |  |  | Analyses with conservative outlier management | | | Analyses with less conservative outlier management | | |
| --- | --- | --- | --- | --- | --- | --- | --- | --- | --- |
| Clinical characteristic |  | ROI | Network measure | Coefficient (95 % CI) | p | BF | Coefficient (95 % CI) | p | BF |
| Suicidal thoughts | past year | medial orbitofrontal | Local efficiency^1^ | 0.28 (0.11, 0.45) | 0.006 | 3.3 |  |  |  |
|  |  | paracentral | W. participation coefficient | 0.34 (0.09, 0.59) | 0.007 | 3.3 |  |  |  |
|  |  | pericalcarine | W. nodal efficiency | -0.51 (-0.80, -0.21) | 0.001 | 15.3 |  |  |  |
|  |  | pericalcarine | Average nearest neighbor degree | -0.47 (-0.73, -0.29) | 0.001 | 16.9 |  |  |  |
| Suicidal thoughts | past month | medial orbitofrontal | W. characteristic path lengths | 0.43 (0.15, 0.70) | 0.002 | 7.5 | 0.43 (0.15, 0.70) | 0.002 | 7.5 |
|  |  | medial orbitofrontal | Characteristic path lengths | 0.45 (0.16, 0.73) | 0.002 | 7.5 | 0.45 (0.16, 0.73) | 0.002 | 7.5 |
|  |  | medial orbitofrontal | Eccentricity | 0.48 (0.20, 0.77) | 0.001 | 14.6 | 0.48 (0.20, 0.77) | 0.001 | 14.6 |
| Suicidal plans | past year | medial orbitofrontal | Eccentricity | 0.44 (0.14, 0.75) | 0.004 | 4.6 |  |  |  |
|  |  | paracentral | W. participation coefficient | 0.34 (0.10, 0.58) | 0.006 | 3.6 |  |  |  |
| Suicide attempt | lifetime | pericalcarine | W. hubness^2^ | 0.66 (0.29, 1.02) | <.001 | 3.1 |  |  |  |
| Suicide attempt | past year | medial orbitofrontal | Hubness | -0.30 (-0.49, -0.08) | 0.006 | 4.5 | -0.33 (-0.53, -0.13) | 0.001 | 16.3 |
| Suicide attempt | past year | medial orbitofrontal | W. hubness |  |  |  | -0.28 (-0.49, -0.08) | 0.008 | 3.3 |
|  |  | pericalcarine | Hubness^3^ | 0.69 (0.31, 1.07) | <.001 | 3.1 |  |  |  |
|  |  |  |  |  |  |  |  |  |  |
| Acts of NSSI | lifetime | paracentral | Degree | -0.31 (-0.53, -0.10) | 0.004 | 4.7 | -0.31 (-0.53, -0.10) | 0.004 | 4.7 |
| Acts of NSSI | past month | medial orbitofrontal | Transitivity | 0.22 (0.08, 0.35) | 0.002 | 8.4 | 0.22 (0.08, 0.35) | 0.002 | 8.4 |
|  |  | medial orbitofrontal | Local efficiency | 0.22 (0.07, 0.37) | 0.005 | 4.5 | 0.22 (0.07, 0.37) | 0.005 | 4.5 |
| Acts of NSSI | past week | medial orbitofrontal | Characteristic path lengths^4^ |  |  |  | 0.19 (0.08, 0.31) | 0.001 | 4.8 |
| Acts of NSSI | past week | medial orbitofrontal | Transitivity^4^ |  |  |  | 0.27 (0.08, 0.45) | 0.005 | 8.7 |

Note. CI = confidence interval. W. = Weighted measure: includes information about connection strength (as opposed to unweighted measures, which refer to binary graph (connection absent vs. present)). ^1^ Interaction effect of hemisphere by clinical variable, no main effect of suicidal thoughts. ^2^ Interaction hemisphere by suicide attempt, small negative main effect of clinical variable. ^3^ Interaction hemisphere by suicide attempt, small negative main effect of clinical variable. ^4^ Interaction effect of hemisphere by clinical variable, no main effect of NSSI acts. Conservative outlier management: outliers +-3SD removed. Less conservative outlier management: clinically plausible but high values included, except for one implausible value in NSSI acts past month.

**Supplementary Figure 1.** Associations between graph-based measures of regional functional connectivity and clinical characteristics in patients engaging in NSSI


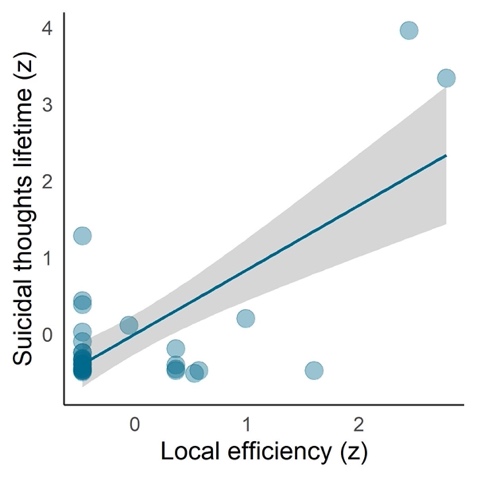

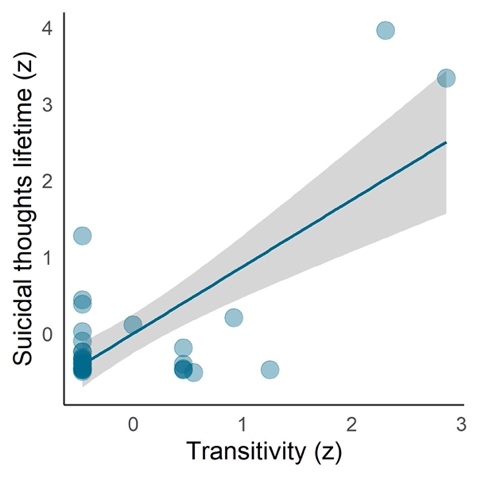

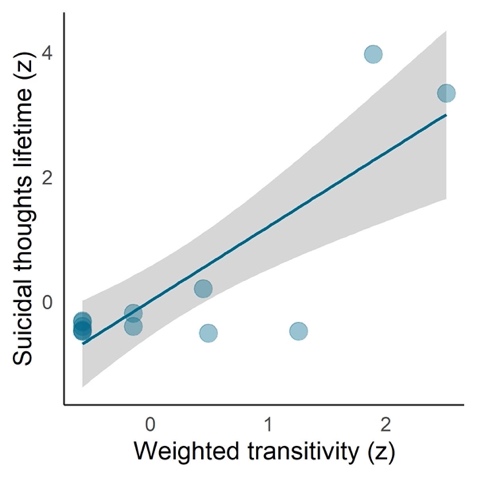


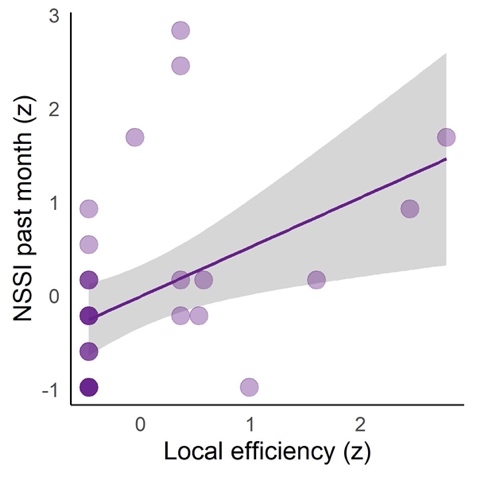

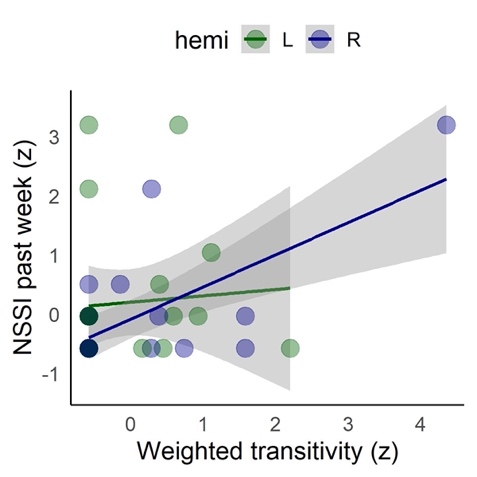

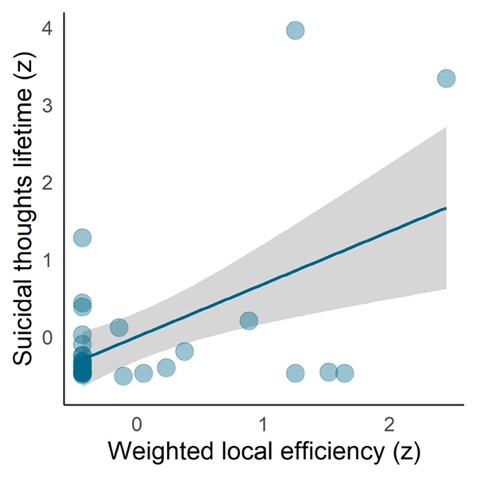


Note: Results from models with BF (= Bayes Factor) > 20 are shown. Each individual measurement is represented by a circle, lines represent linear regression coefficient and grey shaded area represents the standard error. Circles appear darker when multiple measurements are overlaid.
